# Shining a Light on Overnight Education: Hospitalist and Resident Impressions of the Current State, Barriers, and Methods for Improvement

**DOI:** 10.7759/cureus.2939

**Published:** 2018-07-06

**Authors:** Sara N Sani, Emily Wistar, Lien Le, David Chia, Lawrence A Haber

**Affiliations:** 1 Medicine, University of California San Francisco, San Francisco, USA; 2 Gastroenterology, University of California San Diego, San Diego, USA

**Keywords:** overnight education, nocturnist, nightfloat, resident curriculum

## Abstract

Introduction: Restrictions on resident work hours and increased requirements for resident supervision have led to night float rotations overseen by overnight hospitalists (nocturnists). The educational value of night float rotations for residents has traditionally been low and studies have yet to elucidate the optimal role of nocturnists in resident education.

Methods: We performed a cross-sectional survey of all residents within our training program and attending hospitalists in the department of medicine at our three teaching medical centers. Questions sought to investigate the current state of overnight education within an internal medicine residency program, understand barriers to overnight education, and define best practices for nighttime teaching.

Results: Both attending and resident physicians reported low satisfaction with the current state of overnight education, while simultaneously expressing high levels of interest in performing and receiving dedicated nighttime teaching. Attending and resident physicians identified similar barriers to overnight teaching (clinical work, time coordination, provider fatigue) and agreed on the ideal format for overnight didactics (case-based, chalk-talk, 20-minute duration between 10 pm to 2 am).

Conclusion: Our study identifies a desire by both faculty and trainees for increased overnight teaching and offers a simple initial framework for programs to improve overnight housestaff education utilizing nocturnist providers.

## Introduction

Increasing resident supervision mandates and work hour restrictions, enacted in response to the 2011 Accreditation Council for Graduate Medical Education (ACGME) regulations [1], have led to the prevalence of overnight hospitalists (nocturnists) in training environments [2]. While studies have shown nocturnists to be a key resource for improving housestaff well-being and patient care oversight [3-4], their role in formal overnight housestaff education has not been well studied.

Studies demonstrate negative housestaff perceptions of the educational value of night float rotations [5-6]. Recent investigation into improving overnight trainee education has focused on formalizing resident-led curricula [7-8], without considering the role nocturnists can play in enhancing nighttime learning. This is despite evidence that residents find increased educational value to nighttime rotations when working alongside overnight faculty [9-10].

We sought to investigate the current state of overnight resident education at our tri-institutional training program, understand barriers to nighttime teaching, and elicit best practices to guide programs developing overnight resident curriculum utilizing nocturnist providers.

## Materials and methods

The study was conducted at a tertiary care medical center, public safety-net hospital, and Veteran Affairs hospital, all associated with the University of California, San Francisco Internal Medicine Residency Program. There are 186 residents in the training program. First-year residents work a minimum of one week of night float consisting of overnight cross-coverage of patients admitted to the general medicine service. Second and third-year residents complete four weeks on night float rotations, assisting interns with cross-coverage and admitting general medicine patients.

At each hospital, there is a minimum of one nocturnist present every evening. The nocturnist staffs overnight admissions to the intensive care unit, discusses newly admitted step-down patients, and provides supervision of care for any patients undergoing a major change in clinical status. The nocturnist also covers direct care services, performs medicine consults, and admits patients in rotation with housestaff. Within the training program, no overnight educational curriculum or explicit instruction for overnight teaching currently exists.

Three full-time nocturnists, one from each of our three medical centers, developed survey content based on study goals, available literature, and personal experience. Surveys were piloted on a select number of residents and faculty for content and ease of use, with questions further refined in works in progress meetings.

We e-mailed surveys to all residents and attending hospitalists at each training site. We excluded residents who had not worked a night shift in the past year and hospitalists who had not worked a night shift in the past five years. Informed consent was embedded within the e-mail survey request. We did not collect unique identifiers from respondents and no incentive was offered for completion of the survey. Surveys were composed of Likert-style questions as well as free text responses. Survey data were anonymous and downloaded to a database (Qualtrics, Provo, UT) by a third party. Statistical tests of significance were performed using Fisher’s exact test and t-test. The study was approved by the medical center's Institutional Review Board.

## Results

Respondents

There were 64 hospitalist and 42 resident survey respondents (response rate of 34% and 23%, respectively). 19 hospitalists were excluded based on study criteria. Nocturnist shifts were primarily staffed by junior faculty with a mean of 3.3 years of attending experience (range 0-13 years), performing 30.7 shifts per academic year (range 1-110 shifts). All years of medicine residency training were represented (PGY-1 36%, PGY-2 45%, PGY-3 21%).

Current state and barriers

Both attending and resident physicians reported low levels of satisfaction with the current state of overnight education (24% and 12% respectively, p=0.17), yet high levels of interest in performing and receiving dedicated nighttime teaching (67% and 78%, p=0.34). We found a misperception of disinterest between the groups with 68% of hospitalists reporting perceived resident disinterest in receiving overnight teaching, while only 33% of residents self-reported disinterest (p=0.005). Similarly, 71% of residents reported perceived attending disinterest in delivering teaching, while only 29% of hospitalists self-reported disinterest in delivering teaching (p<0.001). Attendings and residents identified similar barriers to overnight teaching including clinical work (84% and 86%, respectively), difficulty coordinating meeting time (76% and 71%), and provider fatigue (40% and 54%); there was no significant difference in responses between the two groups.

Ideal overnight practices

Residents responded that the factors that would most facilitate nighttime teaching were protecting housestaff from clinical duties (57%), setting explicit expectations that teaching occur nightly (45%), and assigning a designated time slot for teaching (36%). Faculty responses were similar, except that hospitalists and residents disagreed over the benefit of protecting trainees from clinical duties to facilitate teaching (33% and 57% respectively, p=0.03).

When queried about the ideal model for overnight discrete teaching sessions, hospitalist and resident respondents agreed that ideal overnight didactics should be case-based (78% and 63%) chalk talks (58% and 71%) of approximately 20 minutes duration (18.4 and 21.5 minutes). Nearly all hospitalists and residents felt that overnight teaching should occur between the hours of 10:00 pm to 2:00 am. There were no statistically significant differences in responses between groups. The above findings, in addition to the six overlapping nighttime teaching topics that both groups reported as high-yield are summarized in Table 1.

**Table 1:** Recommended night curriculum structure

Structure	Respondent Recommendations
Format	Case-based chalk-talk
Duration	20 minutes
Timing	10:00 pm - 2:00 am
High yield topic 1	Codes and rapid responses
High yield topic 2	Cross-cover and common night calls
High yield topic 3	Overnight emergencies
High yield topic 4	Hypoxia and respiratory distress
High yield topic 5	Sepsis and shock
High yield topic 6	Chest pain and myocardial infarction

## Discussion

Our study contributes to a limited body of literature on overnight resident education and is the first, to our knowledge, to investigate perspectives on the role of overnight hospitalists in nighttime didactics. Similar to prior studies, we found low satisfaction with current overnight education among both hospitalist and resident respondents. Our findings, however, dispel the belief that attending and resident physicians are disinterested in receiving or providing nighttime teaching.

In 2014, Hanson et al. called for the development of skilled nighttime educators to realize the unique educational potential of night shifts [11]. Our results speak to the potential of nocturnists in training environments to act as teaching attendings. Even with brief contact, formalizing nocturnist involvement in overnight didactics may present an opportunity for junior faculty to engage with trainees in an educational capacity, foster job satisfaction, and obtain teaching evaluations necessary for academic advancement. Accordingly, junior faculty who perform overnight shifts may benefit from additional teaching training and programs should consider teaching skills as a unique factor when evaluating nocturnist hires.

Our findings reinforce prior reports on barriers to maintaining overnight teaching, namely provider fatigue and completing clinical duties [12]. Clinical needs supersede didactics, especially at night when physician staffing may be reduced. This may account for resident and nocturnist perceptions of lack of interest in receiving and delivering overnight education. It may also be possible that a lack of clear expectations on the part of hospitalists or residents, around the importance of teaching at night or how best to incorporate education into often unstructured overnight clinical hours, may also lead to perceived disinterest. The simplest intervention proposed by our respondents to increase the frequency of night teaching is providing the clear expectation to all overnight providers that teaching will occur.

Our data present relatively granular recommendations for programs to build an overnight curriculum. We found agreement between both faculty and trainees that nighttime sessions should be brief, case-based, chalk talks, occurring between 10 pm and 2 am. Our high-yield teaching topics overlap with prior literature [8], but have the added benefit of perspective from trainees at varying levels of seniority in addition to that of practicing overnight faculty.

There are several limitations to our study. First, the findings represent the experience of internal medicine housestaff within a single residency program and may not be reflective of other programs. The experience of faculty respondents does represent that of three distinct hospitals, though may not be representative of experiences outside of our academic program. Second, we had low response rates from trainees, though not dissimilar absolute numbers compared with similar studies [9]. Finally, we solicited perceptions and preferences of residents and faculty members with regard to current state, barriers, and ideal practices. Whether these preferences would be well received in practice has yet to be determined.

## Conclusions

Our study underscores a desire for increased overnight teaching on the part of both learners and faculty. Future steps will focus on building overnight curricula delivered by nocturnists using evidence-based structure and topics, defining measures of success for overnight education, and examining teaching skills of overnight faculty.
